# Cumulative Live Birth Rate in Patients With Thin Endometrium: A Real-World Single-Center Experience

**DOI:** 10.3389/fendo.2020.00469

**Published:** 2020-09-04

**Authors:** Zhiqin Bu, Linli Hu, Xinhong Yang, Yingpu Sun

**Affiliations:** Henan Province Key Laboratory for Reproduction and Genetics, Reproductive Medical Center, The First Affiliated Hospital of Zhengzhou University, Zhengzhou, China

**Keywords:** cumulative live birth rate, thin endometrium, infertility, IVF, outcome

## Abstract

**Background:** Studies have shown that patients with a thin endometrial thickness (EMT < 7 or 8 mm) during IVF/ICSI tend to have adverse pregnancy outcomes, and this has caused much anxiety to both patients and physicians when confronted with a thin EMT.

**Method:** From January 2015 to December 2018, patients with a thin EMT < 7 mm on the day of hCG administration during their first GnRH agonist IVF/ICSI cycle were included. According to the hysteroscopy results, patients were classified into totally normal (Group A), normal with a specific abnormality (Group B), and adhesion before transfer (Group C).

**Result:** For the 245 patients included, approximately 60% of the thin EMT cases were the result of an intrauterine operation. CLBR was 35.45% (67/189) in this group of patients. In regard to CLBR, there were significant differences among these three uterus condition groups irrespective of the number of oocytes retrieved (28.57 vs. 10.00 vs. 4.76%, *P* = 0.12 in oocyte ≤5; 61.36 vs. 44.67 vs. 23.63%, *P* = 0.00 in oocyte >5). In binary logistic regression analysis, age (OR = 0.09, *P* = 0.03), number of embryos available (OR = 1.71, *P* = 0.00), and uterine condition (OR = 6.77, *P* = 0.00 for group A; OR = 2.55, *P* = 0.04 for group B; Reference = group C), were significantly associated with CLBR. However, EMT and endometrial pattern had no impact on CLBR.

**Conclusion:** An intrauterine operation was the main reason for a thin EMT. Thin EMT patients with a normal uterine cavity and endometrium had a significantly better CLBR compared with those with adhesions before transfer.

## Introduction

For decades, the relationship between endometrial thickness (EMT) and *in vitro* fertilization/intracytoplasmic sperm injection (IVF/ICSI) outcomes has always been a controversial topic (1–3). To date, most studies have shown that patients with a thin EMT (<7 or 8 mm) during IVF/ICSI tended to have adverse pregnancy outcomes, and this has caused much anxiety to both patients and physicians when confronted with a thin EMT. Thus, it is common to see canceled embryo transfer cycles due to a thin EMT in daily practice.

However, why does a thin EMT occur during IVF/ICSI treatment even with superphysical estrogen stimulation? What are the real chances for this group of patients to achieve a live birth, which is the most meaningful outcome from the patient's perspective? According to previous literature, the incidence of thin EMT was only around 5% in all patients undergoing IVF/ICSI treatment (4–6). So few studies have focused on this particular, small group of patients. Among infertility physicians, it is known that most thin EMT cases are associated with uterine lesions, such as adhesions, fibroids, tuberculosis, etc., while a few others apparently have no reason for the condition. In addition, even with a thin EMT, the endometrial pattern is totally normal in some cases, while a heterogeneous echo/uterine cavity fluid is always present in others. Thus, we hypothesized that when a thin EMT is presented, the reasons for this phenomenon are heterogeneous, and pregnancy outcomes are better in patients without uterine abnormalities.

The aims of the current study were to explore the reasons for a thin EMT (<7 mm on the day of human chorionic gonadotropine, hCG administration) during IVF/ICSI treatment, and to compare the cumulative live birth rates in different groups according to uterus condition before embryo transfer.

## Materials and Methods

Data in this study were from the Clinical Reproductive Medicine Management System/Electronic Medical Record Cohort Database (CCRM/EMRCD) in Reproductive Medical Center, First Affiliated Hospital of Zhengzhou University. Written informed consent was obtained from all patients before IVF treatment for physicians collecting basic information and treatment data. This study has been approved by the Institutional Review Board (IRB) of First Affiliated Hospital of Zhengzhou University.

This retrospective cohort study included patients undergoing the first IVF/ICSI treatment cycle from January 2015 to December 2018. Only patients with a “persistent thin endometrium” were included, which means not only EMT < 7 mm on the day of hCG administration, but also that the EMT never reached 7 mm during the following treatment cycles. In addition, as patients with gonadotropin-releasing hormone (GnRH) antagonist protocol and mild stimulation protocol, which are for poor ovarian responders in our center, do not have enough time for Gn stimulation, thus only GnRH agonist treatment protocols were included.

For each patient, the diagnosis and history of infertility were carefully reviewed by at least two of the authors. Endometrium preparation for all patients included in this study was Estrogen-progesterone protocol. High dose of Progynova, 8 mg/day was used from the beginning. Aspirin (100 mg/day), Viagra (25 mg/day), and Growth hormone: 4.5 IU/day for 5 days) were also added from the day of progesterone administration to the day of serum hCG test. An intrauterine operation was defined as a surgery conducted via the uterine cavity. In this study, it mainly included artificial abortion, cesarean section, myomectomy, polypectomy, etc. However, patients with only a history of hysteroscopy or hysterosalpingography without an operation were excluded from the intrauterine operation group.

Each patient included underwent routine hysteroscopy examination before the commence of treatment. According to the hysteroscopy results, patients were classified into totally normal (group A), normal-with a specific abnormality (group B), and adhesion before transfer (group C) group. **Totally normal:** The shape of the uterine cavity and endometrium were normal, with no history of uterine adhesions or endometrial abnormalities. **Normal-with a specific abnormality:** The shape of the uterine cavity was normal, no surgery was needed following the hysteroscopy examination. However, patients in this group were reported to have a clear history of uterine adhesions, endometritis (confirmed by an endometrial biopsy), endometrial tuberculosis (confirmed by an endometrial biopsy), or adenomyosis and fibroids (confirmed by ultrasound), etc. **Adhesion before transfer:** Adhesions were found in this group of people, and intrauterine adhesion separation was performed. Hysteroscopy images of these three groups are shown in Supplemental Figure 1.

### Statistical Analysis

Since the number of oocytes retrieved per cycle was an crucial parameter for live birth, patients were firstly divided into two groups according to the number of oocytes retrieved (poor responders: oocyte retrieved ≤5; non-poor responders: oocyte retrieved >5). After stratified by the oocyte number, the patient basic parameters, and cumulative live birth rate per oocyte retrieval cycle in these three different uterus condition groups were compared using the One way ANOVA/Cruskal-Wallis, or Chi-square test, as appropriate. Binary logistic regression analysis was performed to explore the association between factors and cumulative live birth rate in all 189 patients. Statistical analysis was carried out using the Statistical Program for Social Sciences (SPSS Inc., Version 21.0, Chicago, IL, USA). In all cases, *P* < 0.05 was considered to be statistically significant.

## Results

**Figure 1:**
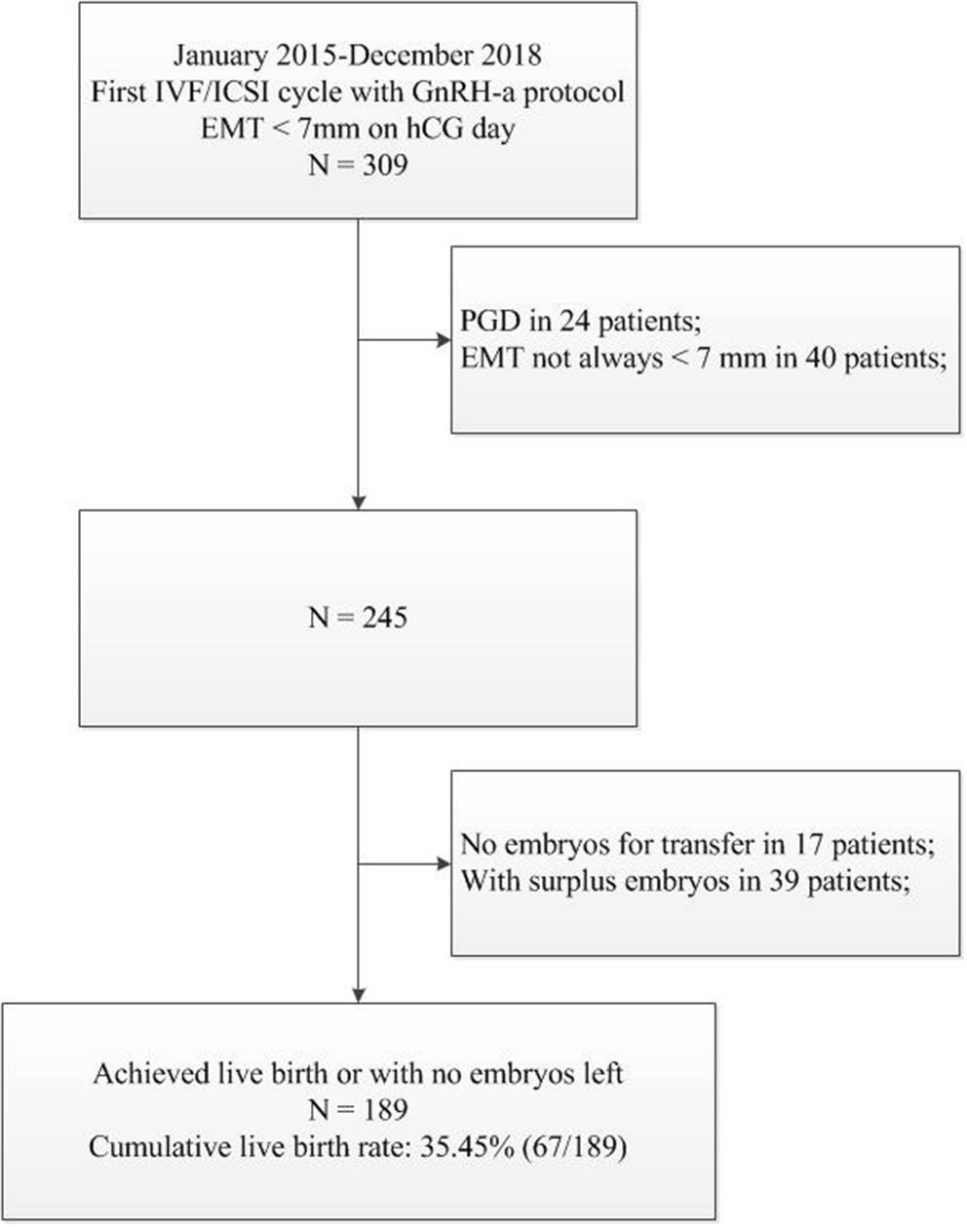
Study Flow Chart.

From January 2015 to December 2018, there were 309 patients with EMT <7 mm on day of hCG administration with their first GnRH agonist IVF/ICSI cycles. Among these, 24 PGD patients and 40 patients (EMT ≥ 7 mm in the following treatment cycles) were excluded (Figure 1).

**Figure 2:**
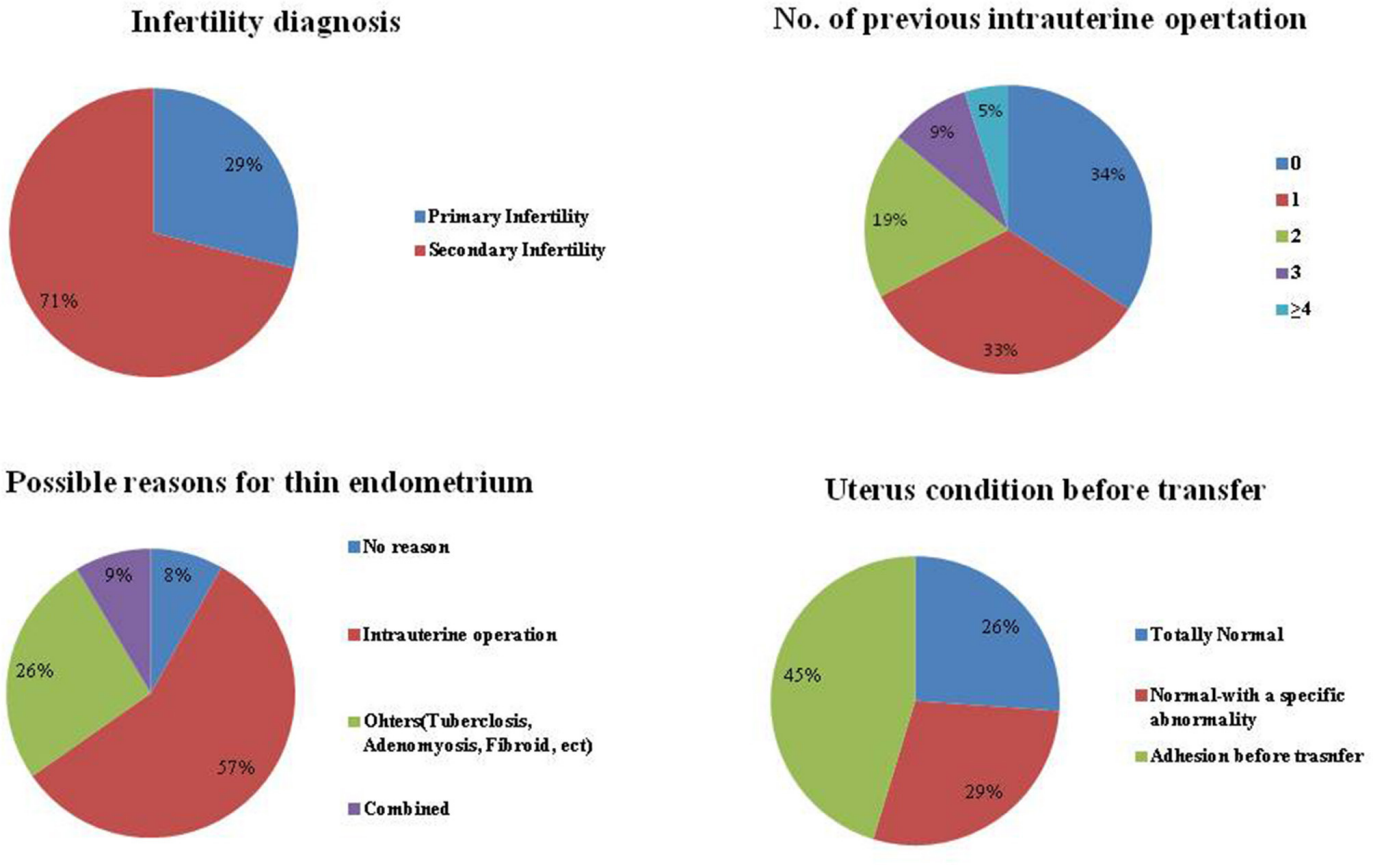
Detailed information for patients included.

For the 245 patients with a “persist thin endometrium,” their detailed information is shown in Figure 2. We found that 71% of these patients were diagnosed with secondary infertility, and 66% of these patients had a history of an artificial abortion. In addition, no reason was found for a thin EMT for only 8% of the patients. Approximately 60% cases of thin EMT appeared to have been caused by an intrauterine operation. A known uterine abnormality (endometritis, endometrial tuberculosis, adenomyosis, fibroids, or with a clear history of uterine adhesions, etc.) were observed for 26% of these patients. Moreover, if all 245 patients were divided according to uterus condition before embryo transfer, the proportion of totally normal, normal-with a specific abnormality, and adhesion before embryo transfer, were 26, 29, and 45%, respectively.

Among these 245 patients with thin EMT, no embryos were available for transfer in 17 patients, and 39 patients (including 3 ongoing pregnancies) did not achieve live birth until August 2019 but with surplus embryos. Thus, the cumulative live birth rate was calculated for 189 patients either with a reported live birth or no embryos left from this oocyte retrieval cycle. In total, 67 achieved a live birth, a cumulative live birth rate of 35.45% (67/189).

As for the patient basic parameters and IVF/ICSI outcome, as shown in Table 1, the patient age, BMI, number of oocytes retrieved, as well as the number of embryos available for transfer, were comparable between the three uterus condition groups for both poor and non-poor responders. However, for non-poor responders in group A and group B with a normal uterine cavity, the mean EMT on the day of hCG administration was significantly thicker (5.84 vs. 5.48 vs. 5.38 mm; *P* = 0.01), and the proportion of Triple-line endometrium was higher (81.82 vs. 57.78 vs. 27.27%; *P* = 0.00), compared with those in patients from group C with adhesions before transfer. This situation was similar in poor responders, although the difference was not statistically significant.

**Table 1:** Basic parameters and treatment outcomes in patients with different uterine condition stratified by number of oocytes retrieved.

	**No. of oocytes retrieved ≤5 Poor responders**				**No. of oocytes retrieved >5Non-poor responders**			
	**Group A**	**Group B**	**Group C**	***P***	**Group A**	**Group B**	**Group C**	***P***
No. of patients	14	10	21		44	45	55	
Age (years)	36.86 ± 3.84	35.80 ± 5.63	36.48 ± 4.50	0.86	33.61 ± 4.91	33.76 ± 5.40	33.49 ± 4.83	0.97
BMI (Kg/m^2^)	22.85 ± 2.55	23.91 ± 3.46	22.39 ± 3.34	0.46	23.01 ± 2.97	22.90 ± 3.06	22.52 ± 2.81	0.68
No. of oocytes retrieved	3.36 ± 0.93	3.40 ± 1.27	3.67 ± 1.32	0.72	11.36 ± 5.60	12.31 ± 4.18	13.09 ± 5.36	0.25
EMT (mm)	5.79 ± 0.43	5.80 ± 0.42	5.55 ± 0.59	0.29	5.84 ± 0.56^*^^#^	5.48 ± 0.76^*^	5.38 ± 0.84^#^	0.01
EMP (%)	71.43% (10/14)	60.00% (6/10)	33.33% (7/21)	0.07	81.82% (36/44)^*^^#^	57.78% (26/45)^*^^&^	27.27% (15/55)^#^^&^	0.00
No. of embryos available	1.86 ± 0.54	2.00 ± 0.67	1.90 ± 0.77	0.88	4.25 ± 3.04	4.42 ± 1.76	4.18 ± 2.21	0.08
No. of embryos left	0.14 ± 0.55	0.00 ± 0.00	0.05 ± 0.22	0.55	1.68 ± 3.13	1.07 ± 1.71	0.96 ± 2.17	0.30
TTLB	1.00 ± 0.00	1.00 ± 0.00	1.00 ± 0.00	–	1.11 ± 0.32^#^	1.50 ± 0.89	1.58 ± 0.79^#^	0.06
CLBR	28.57% (4/14)	10.00% (1/10)	4.76% (1/21)	0.12	61.36% (27/44)^*^^#^	44.67% (21/45)^*^^&^	23.63% (13/55)^#^^&^	0.00

*Group A, Total normal; Group B, Normal-with a specific abnormality; Group C, Adhesion before transfer; BMI, Body mass index; EMT, Endometrial thickness on the day of hCG administration; EMP, ratio of endometrial pattern being triple-line; TTLR, Time of Transfer to achieve a Live Birth; CLBR, Cumulative live birth rate per oocyte retrieve cycle*.

* Group A vs. Group B, P < 0.05;

# Group A vs. Group C, P < 0.05;

& *Group B vs. Group C, P < 0.05*.

In regard to cumulative live birth rate, there were also significant difference among these three groups irrespective of the number of oocytes retrieved (28.57 vs. 10.00 vs. 4.76%, *P* = 0.12 in poor responders; 61.36 vs. 44.67 vs. 23.63%, *P* = 0.00 in non-poor responders). Moreover, for non-poor responders, patients in group C needed more transfer cycles (1.58 transfer cycles) to achieve a live birth compared with patients in group A (1.11 transfer cycles).

In binary logistic regression analysis, the association between well-known parameters (age, number of embryos available) that have an impact on cumulative live birth, EMT and endometrial pattern, as well as uterine condition before transfer, were analyzed (Table 2). It was shown that age (OR = 0.09, *P* = 0.03), number of embryos available (OR = 1.71, *P* = 0.00), and uterine condition (OR = 6.77, *P* = 0.00 for Group A; OR = 2.55, *P* = 0.04 for Group B; Reference = Group C), were significantly associated with cumulative live births. However, both EMT and endometrial pattern on the day of hCG administration had no impact on cumulative live birth in the logistic regression model.

**Table 2:** Factors associated with Cumulative live birth by logistic regression analysis.

	**Cumulative live birth**	
	**Adjusted OR (95% CI)**	***P***
Age (years)	0.09 (0.85–0.99)	0.03
EMT (mm)	1.34 (0.77–2.45)	0.28
Endometrial pattern (triple line: yes/no)	1.25 (0.55–2.83)	0.59
No. of embryos available	1.71 (1.37–2.13)	0.00
Uterine condition		
Total normal	6.77 (2.46–18.61)	0.00
Normal-with a specific abnormality	2.55 (1.11–6.32)	0.04
Adhesion before transfer (reference)	–	–

*EMT, endometrial thickness on the day of hCG administration; OR, odds ratio; CI, confidential interval*.

## Discussion

Since a thin EMT occurs infrequently, the sample size for this group of patients in previous studies was relatively small. To the best of our knowledge, this study was the first one to focus on patients with a thin EMT during IVF/ICSI treatment. Our study showed that in most cases (92% of patients), a thin EMT was associated with at least one possible reason and that the most common reason for a thin EMT was an intrauterine operation. In addition, even with a thin EMT, patients with a normal uterine cavity before embryo transfer had better pregnancy outcomes.

To date, although there are a few dissenting opinions, it has almost reached a consensus that pregnancy outcomes among thin EMT patients were poor compared with those in patients with a normal EMT (1, 7–10). What is more, one retrospective study recently showed that a thin endometrium was associated with a decrease in singleton birth weight of singletons resulting from frozen thawed embryo transfer cycles (11).

In these previous studies, the cut off values for a thin EMT were not the same; however, 7 and 8 mm are two of the most commonly used. Actually, this issue is very common and important in EMT studies. The value of EMT varies dramatically if the methods (trans-vaginal or trans-abdomen) used by the physicians to measure it are not the same. In the current study, even though the EMT was measured by trans-vaginal ultrasound for all patients, it was impossible for all of the patients to be examined by one physician to ensure consistency. To minimize this bias, we only included patients with an EMT that never reached 7 mm during any ultrasound examination in all treatment cycles. In addition, both patients and physicians were very concerned about EMT if confronted with a thin one, and thus the EMT in this study was measured carefully several times for all patients.

In this study, we found that ~60% cases of a thin EMT were associated with an intrauterine operation, especially with artificial abortion, which is consistent with the common sense that an artificial abortion may damage the basal layer of the endometrium and adversely affect cell growth (12, 13). In addition, endometritis caused by unknown infections, and endometrial tuberculosis also interrupt the normal growth of the endometrium (14). Patients included in this study with adenomyosis or fibroids were without uterine cavity involvement. Nevertheless, these lesions may induce uterine morphological changes, damage the local blood supply, and adversely affect the EMT eventually (15, 16).

The thin uterine condition was not always correlated with an intrauterine operation. Severe intrauterine adhesions occurred in patients with only one intrauterine surgery; however, the uterine cavity and endometrium were totally normal in patients with several artificial abortions. Thus, we divided the 189 patients into different groups according to the final uterine condition before embryo transfer and not according to the reasons for a thin EMT.

Why do thin EMT patients always have poor pregnancy outcomes? It was shown in an earlier study that a thin EMT was characterized by high blood flow impedance of the radial artery, poor epithelial growth, and poor vascular development (17). Hysteroscopy images from our study also clearly showed that in cases of a thin EMT, especially in cases with severe adhesions, the endometrium was pale and the blood vessels were sparse, which resulted in insufficient blood supply to the endometrium, and affected the process of embryo implantation and decidualization of the endometrial stromal cells (18).

The treatment of thin EMT mainly includes: hormone therapy (a high dosage of estrogen), vasoactive drugs such as aspirin, intrauterine granulocyte colony stimulating factor (G-CSF) perfusion and stem cell treatment (19, 20). In this study, all thin EMT patients were treated with the standard procedure, which included a high dose of Progynova, aspirin, growth hormone, and Viagra at our center, but with only limited effect in improving EMT.

Interestingly, our study showed that even with a history of uterine adhesion, those with a normal uterine cavity after adhesion separation had better results compared with those who still had the adhesion before transfer. This indicated that trying to separate adhesions and to restore a normal uterine cavity seems to be an effective method for improving pregnancy outcomes in some cases.

Moreover, the logistic regression analysis once again proved that, even in this particular group of patients, the age and number of embryos available for transfer were independent factors associated with the cumulative live birth rate. However, EMT or endometrial pattern had little impact on pregnancy outcome. This is important because among those with a normal uterine cavity and younger patients, even those with a thin EMT, should be aware that they actually have a good prognosis.

Several limitations exist in the current study. First, in the totally normal group, we believed that the uterus was not actually totally “normal.” There must be a reason for a thin EMT, such as a missing history of tuberculosis, an atypical endometritis, or other lesions that we did not see. In addition, we classified patients according to their uterus condition before the first embryo transfer cycle. During the study period, there were a total of 11 miscarriages in these 189 patients, of which 8 cases were from group C, 2 cases from group B and 1 case from group A. However, additional hysteroscopy was performed after each miscarriage, and the uterine condition did not change compared with before.

In summary, our study explored the possible reasons for a thin EMT in IVF/ICSI treatment, and we found that an intrauterine operation was the main reason for a thin EMT. In addition, those with a normal uterine cavity and endometrium had significantly better pregnancy outcomes compared with those with adhesions before transfer. This is important information for patient consultations, and we should be cautious about canceling transfer cycles when confronted with thin EMT cases.

## Data Availability Statement

The raw data supporting the conclusions of this article will be made available by the authors, without undue reservation, to any qualified researcher.

## Ethics Statement

The studies involving human participants were reviewed and approved by Institutional Review Board (IRB) of First Affiliated Hospital of Zhengzhou University. The patients/participants provided their written informed consent to participate in this study.

## Author Contributions

ZB contributed to execution of the study, completed the data analysis, and wrote the article. LH and XY contributed to critically revised the article and approved the final version. YS conceived the study design, contributed to interpretation of the data, critically revised the article, and approved the final version. All authors contributed to the article and approved the submitted version.

## Conflict of Interest

The authors declare that the research was conducted in the absence of any commercial or financial relationships that could be construed as a potential conflict of interest.
