# Supplementary figures and images for: Cumulative Live Birth Rate in Patients With Thin Endometrium: A Real-World Single-Center Experience
Source: Front Endocrinol (Lausanne). 2020 Sep 4;11:469. doi: 10.3389/fendo.2020.00469 (PMC7509444; doi:10.3389/fendo.2020.00469)

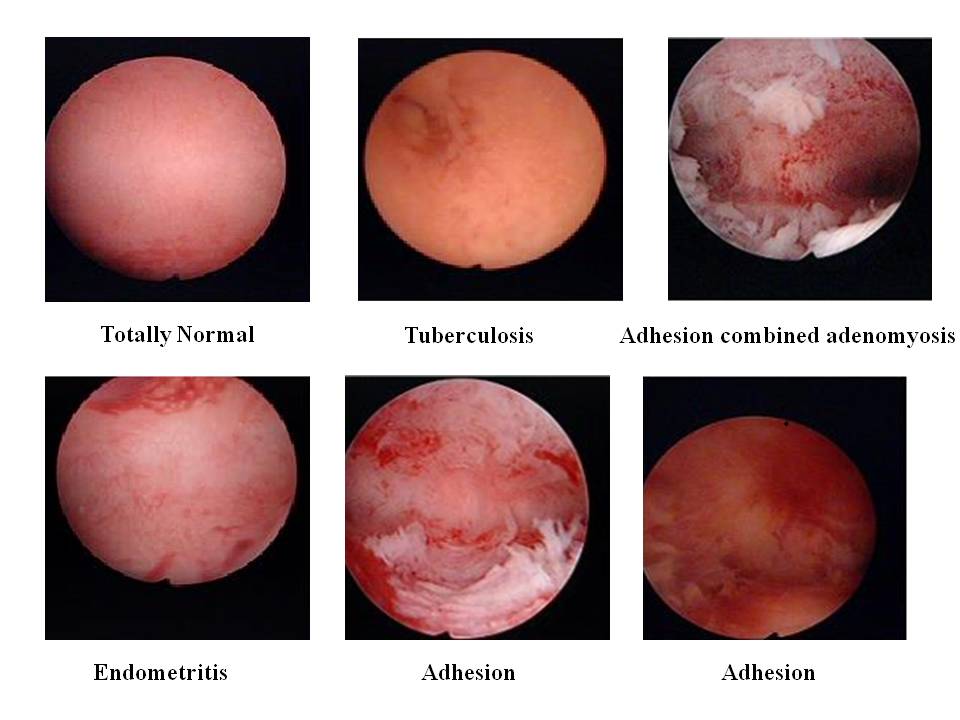

Supplement: Supplementary file 1 [file Image_1.JPEG]
